# Blood Group Rhesus D-negativity and Awareness Toward Importance of Anti-D Immunoglobulin Among Pregnant Women in Bisha, Saudi Arabia

**DOI:** 10.7759/cureus.7044

**Published:** 2020-02-19

**Authors:** Amar Yahia, Elhadi Miskeen, Shahzada K Sohail, Tarig Algak, Saad Aljadran

**Affiliations:** 1 Basic Medical Sciences, College of Medicine, University of Bisha, Bisha, SAU; 2 Obstetrics and Gynecology, College of Medicine, University of Bisha, Bisha, SAU; 3 Laboratory Medicine, King Abdullah Hospital, Bisha, SAU

**Keywords:** rhesus d antigen alloimmunization, blood group rh(d)-negativity, anti-d immunoglobulin, awareness

## Abstract

Background

Rhesus D (RhD) antigen alloimmunization has been a focus of concern for hematologists and obstetricians. It contributes to perinatal morbidity and mortality. The objectives of this study were to assess the awareness of pregnant women toward the clinical importance of blood group Rh(D)-negativity and anti-D immunoglobulin and to determine the prevalence of blood group Rh(D)-negativity among them.

Methods and materials

This cross-sectional study was conducted in a routine antenatal care clinic of King Abdullah Hospital in Bisha, Saudi Arabia from September 2018 to January 2019. The awareness of pregnant women toward the clinical importance of blood group Rh(D)-negativity and prophylaxis with anti-D immunoglobulin was assessed through a self-administered questionnaire. Samples were analyzed for ABO and Rh (D) blood groups using the microplate grouping method. The presence of anti-D alloantibodies was detected by the indirect antiglobulin test. Data were analyzed by IBM SPSS Statistics for Windows, Version 25.0 (Armonk, NY: IBM Corp.). A p-value ≤0.05 was considered statistically significant.

Results

A total of 108 respondents fulfilled the inclusion criteria and completed the survey. Forty-five pregnant women (41.7%) were observed to be aware of the blood group Rh(D)-negativity and anti-D immunoglobulin issue. The prevalence of the negative blood group was 11.1% (12/108). Awareness was found to be significantly associated with age, education, vaginal bleeding, blood groups, and previous administration of anti-D immunoglobulin (p-value ≤0.05).

Conclusion

The prevalence of blood group Rh(D)-negativity among respondents was found to be comparative with other populations, although the overall awareness was found to be suboptimal (41.7%). Structured health education programs by hematologists and obstetricians are needed to increase awareness and to address women at reproductive age.

## Introduction

Rhesus D (RhD) antigen alloimmunization has been a focus of concern for hematologists and obstetricians due to its significant contribution to perinatal morbidity and mortality as a consequence of hemolytic disease of the fetus and the newborn (HDFN). Rhesus alloimmunization represents an avoidable direct cause for perinatal morbidity and mortality, so health education sittings are needed to increase the awareness of the public about this important issue [1,2]. Screening of pregnant women for blood groups has been well established and widely practiced in the antenatal care clinic. The introduction of anti-D prophylaxis has significantly reduced perinatal deaths from alloimmunization by approximately 100-fold [3,4]. Administration of anti-D immunoglobulin is an effective protective measure in reducing the risk of HDFN [3,5]. The aim of this study was to assess the awareness of pregnant women toward the clinical importance of blood group Rh(D)-negativity and anti-D immunoglobulin at King Abdulla Hospital in Bisha, Saudi Arabia as well as to determine the prevalence of blood group Rh(D)-negativity among pregnant women in the local population.

## Materials and methods

Study design and setting

This cross-sectional study was conducted in a routine antenatal care clinic of King Abdullah Hospital in Bisha from September 2018 to January 2019. This is the main tertiary hospital in Bisha and serves a wide range of the population in 240 villages.

Study population and data collection

A total of 108 pregnant women verbally consented and participated in the study. Awareness of the respondents toward the clinical importance of blood group Rh(D)-negativity and prophylaxis with anti-D immunoglobulin was assessed through a self-administered questionnaire. The questionnaire design and content validity were checked and approved by a research committee at the College of Medicine, University of Bisha. The questionnaire consisted of socio-demographic characteristics, blood group, parity, history of vaginal bleeding in early pregnancy, previous administration of anti-D immunoglobulin, and variables that assessed respondents' awareness toward the clinical importance of blood group Rh(D)-negativity and anti-D immunoglobulin. The questionnaire was translated into Arabic and subjected to a process of forward and backward translation. The study was approved by the institutional review board of King Abdulla Hospital.

Method of testing blood group Rh (D)

Peripheral blood was drawn into ethylenediaminetetraacetic acid-containing vacutainer tubes. Samples were analyzed for ABO and Rh (D) blood groups using the microplate grouping method. The presence of anti-D alloantibodies was detected by the indirect antiglobulin test, which involved the incubation of standard cells with the test serum at 37°C for 30 minutes and then reacting with anti-human globulin to bring about agglutination of the red cells in positive samples.

Statistical analysis

Data were analyzed using IBM SPSS Statistics for Windows, Version 25.0 (Armonk, NY: IBM Corp.). Categorical variables were described using frequencies and percentages. A univariate analysis was conducted using a chi-squared test for these variables. A p-value of ≤0.05 was considered statistically significant.

## Results

**Figure 1:**
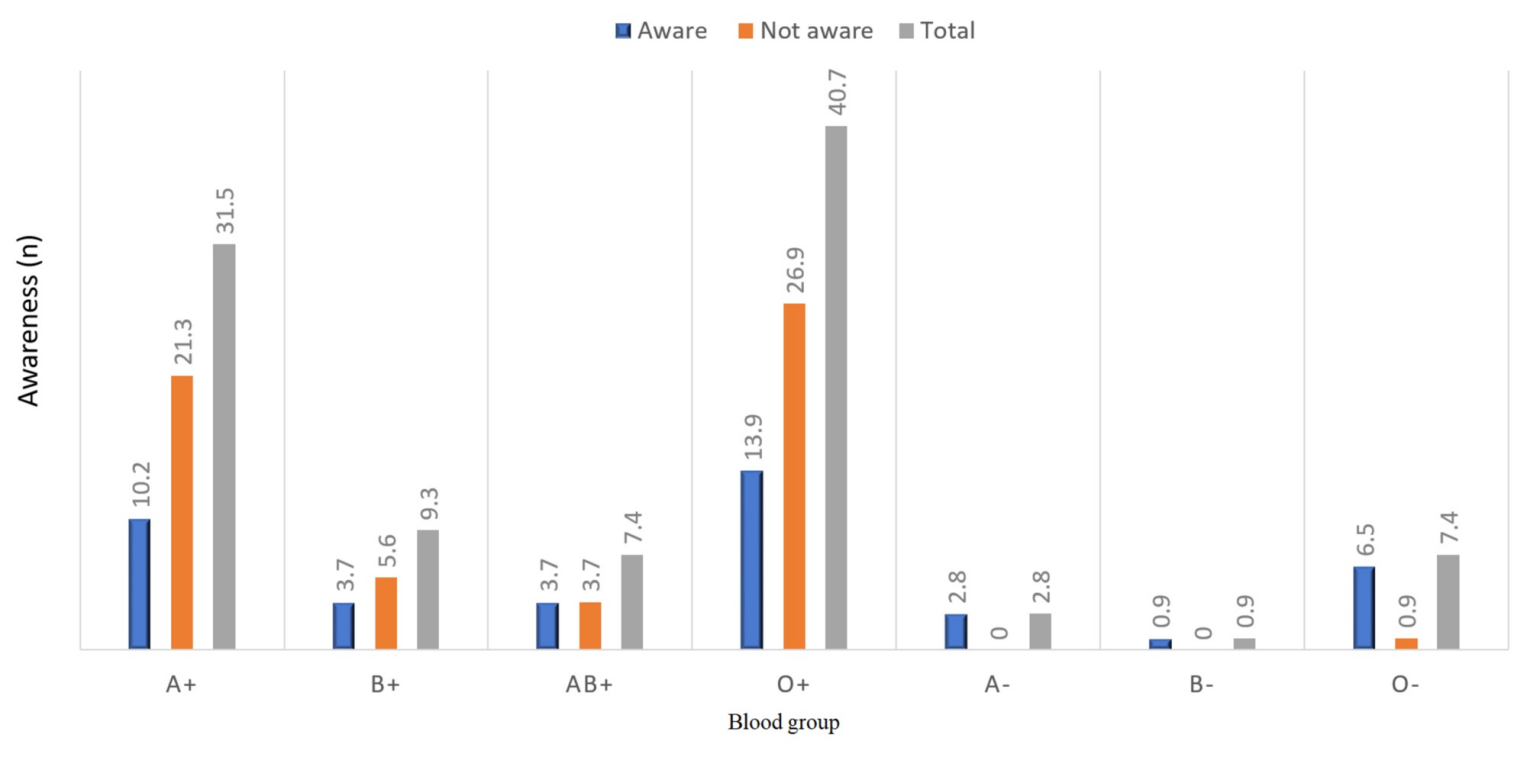
Distribution of blood groups and its association with the awareness (N=108)

**Figure 2:**
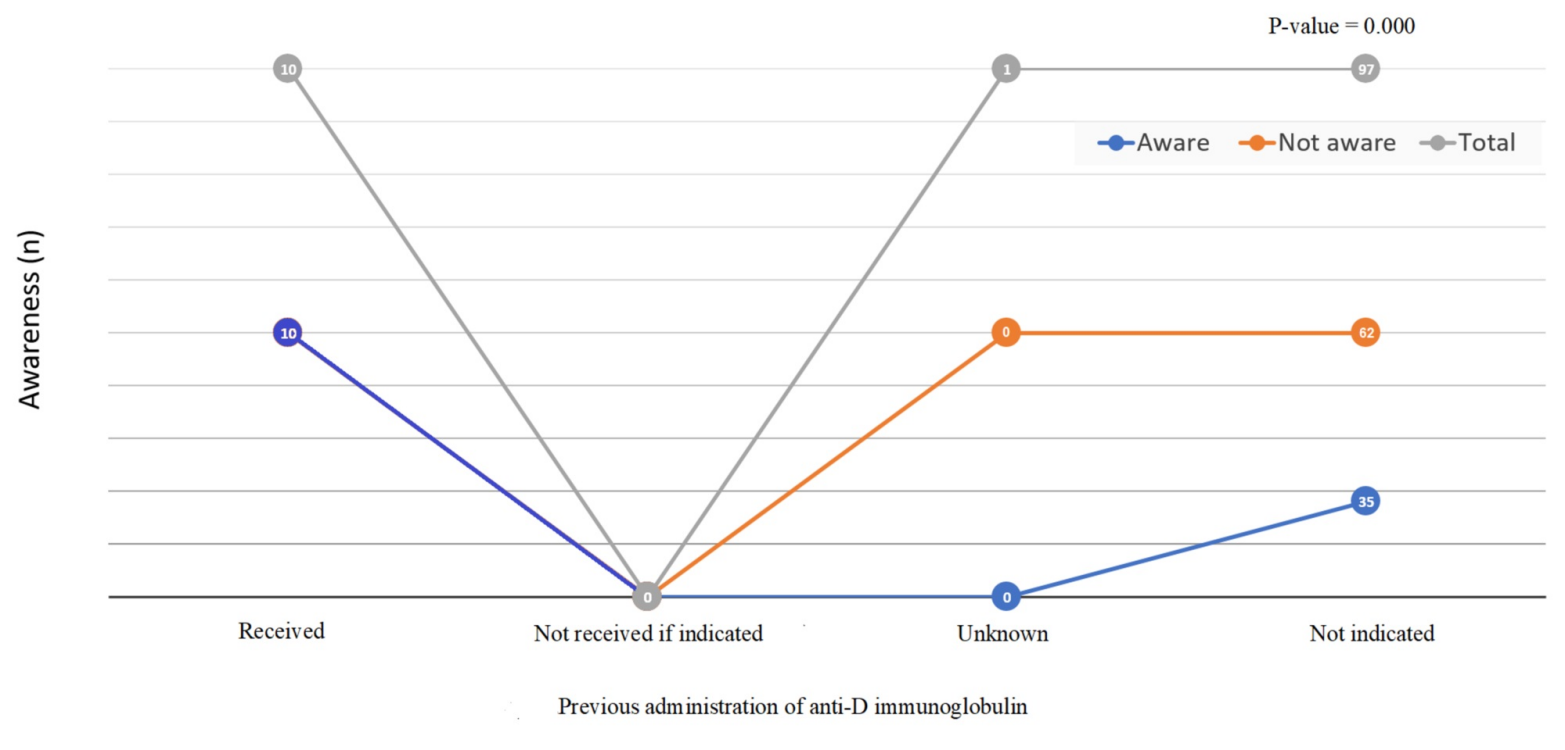
Previous administration of anti-D immunoglobulin and its association with the awareness (N=108)

A total of 108 respondents fulfilled the inclusion criteria and completed the survey. Most respondents-71/108 (65.7%)-were within the age group of 18-35 years. Only one-quarter of respondents-27/108 (25.0%)-had achieved a university-level education. Forty-five pregnant women (41.7%) were observed to be aware of the blood group Rh(D)-negativity and anti-D immunoglobulin issue. This study found a statistically significant association of this awareness with increasing age and education level of the respondents with p-values of 0.035 and 0.001, respectively. In this study, 77/108 (71.3%) of the respondents were parous women. About one-third of those parous women-37/108 (34.3%)-have experienced vaginal bleeding during pregnancy, which showed a statistically significant association with their awareness (p-value=0.000; Table 1). The prevalence of the negative blood group was 11.1% (12/108). The most common blood group among the study population was O positive, 44/108 (40.7%). The distribution of other blood groups is shown in Figure 1. The association between blood group type and awareness showed a statistically significant result (p-value=0.001). Among the study population with negative blood groups, the majority-10/11 (91.6%)-had received anti-D immunoglobulin; however, one patient was not sure about receiving the anti-D immunoglobulin. A strong association was found between the previous administration of anti-D immunoglobulin and awareness (p-value=0.000; Figure 2).

 

**Table 1:** Characteristics of the study population and association with their awareness (N=108)

Characteristics	Aware	Not Aware	Total (%)	P-value
Age group in years				0.035
Younger than 18	6 (5.6%)	10 (9.3%)	16 (14.8%)	
18-35	25 (23.2%)	46 (42.6%)	71 (65.7%)	
Older than 35	14 (13.0%)	7 (6.5%)	21 (19.4%)	
Educational level				0.001
University	25 (23.2%)	2 (1.9%)	27 (25.0%)	
Secondary	13 (12.0%)	30 (27.8%)	43 (39.8%)	
Primary	7 (6.5%)	31 (28.7%)	38 (35.2%)	
Parity				0.105
Primigravida	8 (7.4%)	23 (21.3%)	31 (28.7%)	
Parous	37 (34.3%)	40 (37.0%)	77 (71.3%)	
Vaginal bleeding in early pregnancy				0.000
Yes	29 (26.9%)	8 (7.4%)	37 (34.3%)	
No	16 (14.8%)	55 (50.9%)	71 (65.7%)	

 

## Discussion

This study provided valuable results regarding important daily practice issues: blood group Rh(D)-negativity and the importance of anti-D immunoglobulin. Prevalence, awareness, and importance of anti-D immunoglobulin were assessed in the main tertiary hospital in Bisha, Saudi Arabia. The prevalence of negative blood groups among pregnant women in this study was 11.1%. The prevalence of Rh(D)-negative blood groups varies in ethnic populations, with approximately 15.8% of Caucasians, 8% of Blacks, and 1% of Asians [6]. The prevalence of negative blood group revealed by this study is more than that reported by the Netherlands (8.9%), a previous Saudi study (7.5%), Nigeria (4.5%), and Oman (7.3%), and it is less than that reported by Pakistan (13.6%) [6-10]. This finding appears to be comparable with other ethnic groups. The antenatal administration of anti-D immunoglobulin prophylaxis is necessary for all Rh(D)-negative women [11]. The overall awareness of the blood group Rh(D)-negativity and the clinical importance of anti-D immunoglobulin was found to be suboptimal (41.7%) in our study; however, a statistically significant association was found among awareness regarding this important clinical issue and blood group type, previous administration of anti-D immunoglobulin, age, educational level, and previous experience of vaginal bleeding. Our study showed that the highest level of awareness was associated with the previous reception of anti-D immunoglobulin (100%) and among women with negative blood groups (91.6%). This finding might be due to their previous experience with the same issue. This study also reflected increased awareness among older women and higher education categories. The level of awareness among our study population was higher compared to that of Nigeria, where awareness of maternal-fetal blood incompatibility of expectant mothers was reported to be 39% [12]. A previous study from Saudi Arabia showed that only 38% of the studied mothers had awareness about Rh(D) incompatibility, 68.5% had awareness about anti-D immunoglobulin, and 51% had awareness about the time of administration of anti-D immunoglobulin [13]. A study in Singapore reported that only 49.1% of women had adequate awareness and 40.0% and 10.9% of the participants had inadequate and poor knowledge, respectively [14].

## Conclusions

The prevalence of blood group Rh(D)-negativity among respondents was found to be comparable with other populations. The overall awareness of the blood group Rh(D)-negativity and the clinical importance of anti-D immunoglobulin was found to be suboptimal in our study. Structured health education programs by obstetricians are needed to increase awareness and to address women at reproductive age.
